# Supplementary material for: Coastal bacteria and protists assimilate viral carbon and nitrogen
Source: ISME J. 2024 Nov 13;18(1):wrae231. doi: 10.1093/ismejo/wrae231 (PMC11629701; doi:10.1093/ismejo/wrae231)
Supplement: Virovory_supplementary_material_wrae231 [file virovory_supplementary_material_wrae231.pdf]

Supplementary material for "Coastal bacteria and protists assimilate viral carbon and nitrogen" by Joaquín Martínez Martínez, David Talmy, Jeffrey A. Kimbrel, Peter K. Weber, Xavier Mayali

Table S1: Experimental design summary

	EhV lysate purification	EhV lysate concentration	Microbial community sampling time	Microbial community size fractions	Incubation volume	EhV addition	Sampling time points	Analyses
**Experiment 1**	<0.45 µm<br>50 KDa	2.2 L to 300 ml	Late summer<br>Incoming tide	<100 µm<br><0.2 µm	500 ml	~25 × 10^6 EhV ml^{-1}	0 h, 12 h, 20 h, 40 h, 27 d, 69 d	flow cytometry (virus and bacteria abundance) nanoSIMS (isotope labeling)
**Experiment 2**	<0.45 µm<br>300 KDa	2.2 L to 300 ml	Early summer<br>Outgoing tide	<100 µm<br><0.1 µm	1000 ml	~30 × 10^6 EhV ml^{-1}	0 h, 12 h, 24 h, 48 h, 8 d	flow cytometry (virus and bacteria abundance) nanoSIMS (isotope labeling) amplicon sequening (16S rRNA + 18SrRNA)
**Experiment 3**	<0.45 µm<br>300 KDa	4.4 L to 150 ml	Late autumn<br>~high tide	<100 µm<br><1.2 µm<br><0.1 µm	250 ml	~30 × 10^6 EhV ml^{-1}	0 h, 12 h, 24 h, 48 h, 4 d, 8 d	flow cytometry (virus and bacteria abundance) nanoSIMS (isotope labeling)

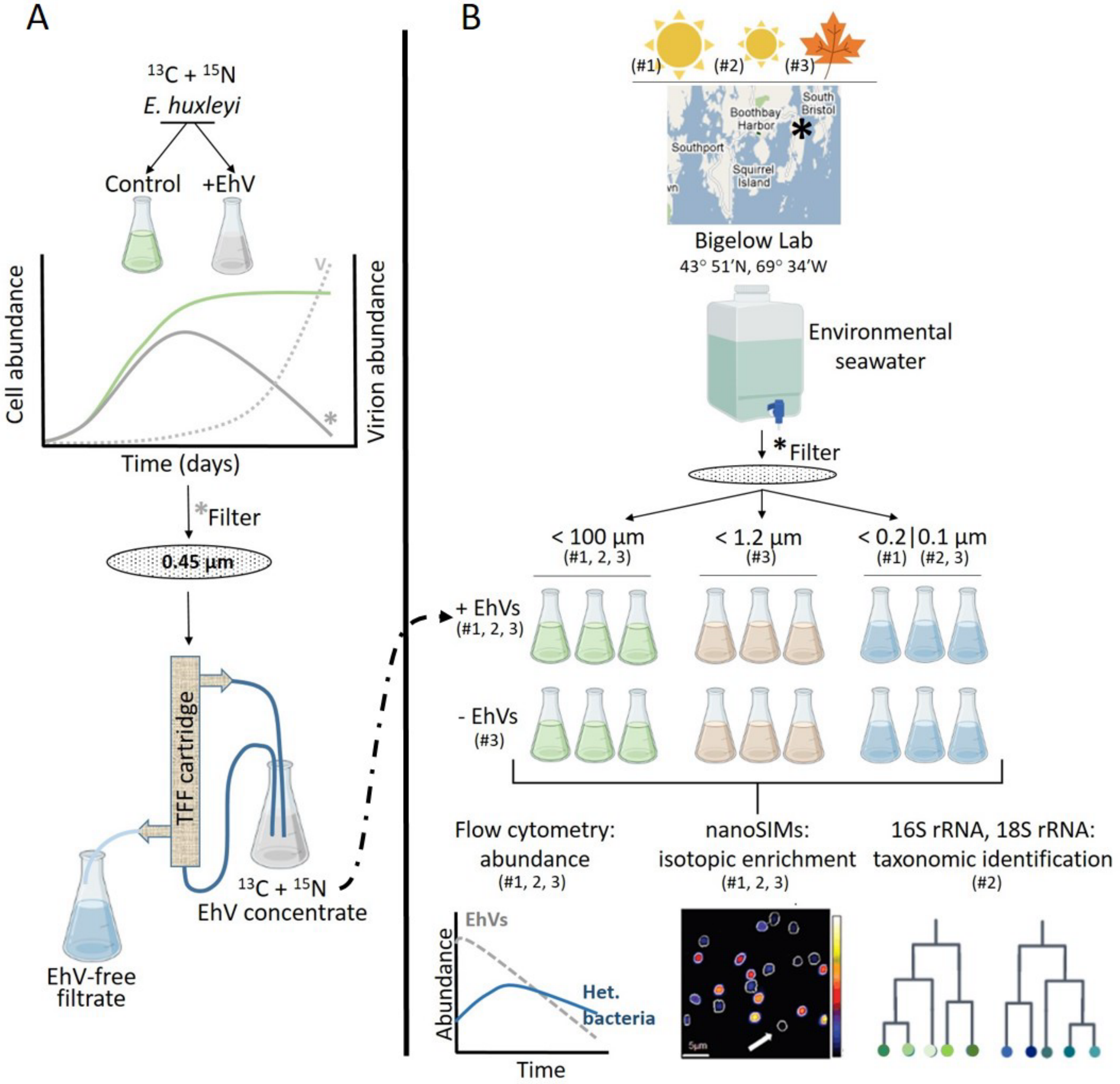

Figure S1: Summary of sampling and experimental design for the three incubations: (#1: long incubation experiment), (#2, high purification experiment), (#3, bacterial fraction experiment)

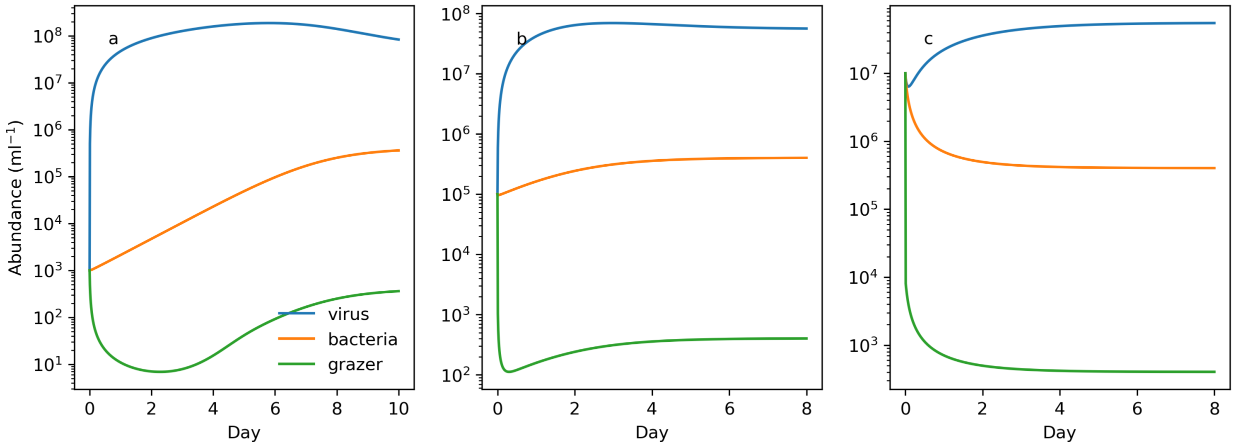

Figure S2: Food web model preliminary simulation ('spin-up') allowing the system to reach equilibrium prior to simulating the bacterial fraction experiment. All model parameter values are provided in Table 1 of the main text. The model converged to the same set of equibrium values regardless of initial conditions, as demonstrated with sensitivities assuming initial abundances for viruses, grazers, and bacteria were a) $1x10^3$, b) $1x10^5$, and c) $1x10^7$ mL^{-1}.

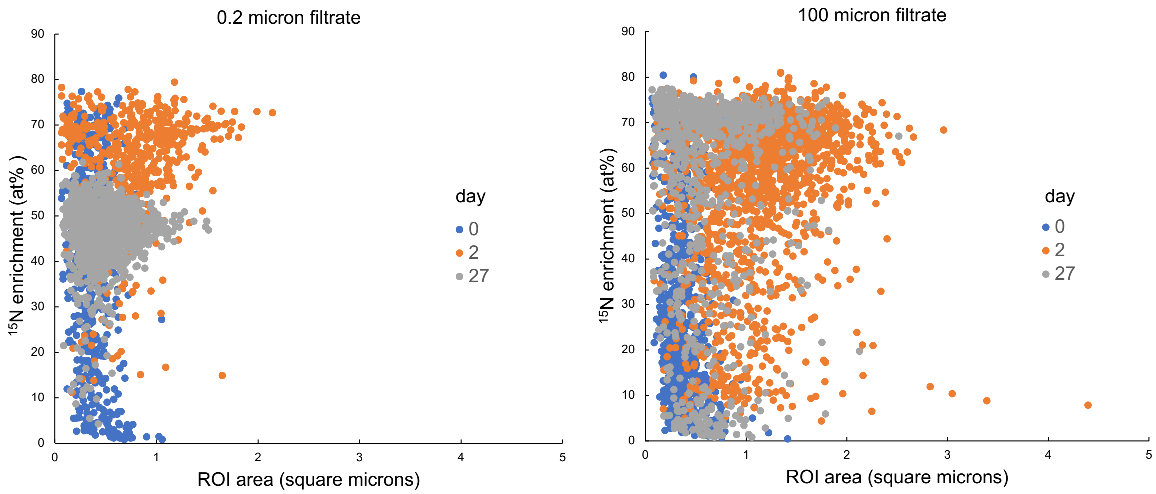

Figure S3: ^{15}N isotopic enrichment of regions of interests (ROIs) of different sizes from the long incubation experiment (#1), collected from nanoSIMS images. ROIs were drawn automatically using the $^{12}C^{14}N^{-}$ images (see figure 1 in main text). These data show that the 100 micron filtrates included larger cells (with larger ROI areas) than the 0.2 micron filtrates.

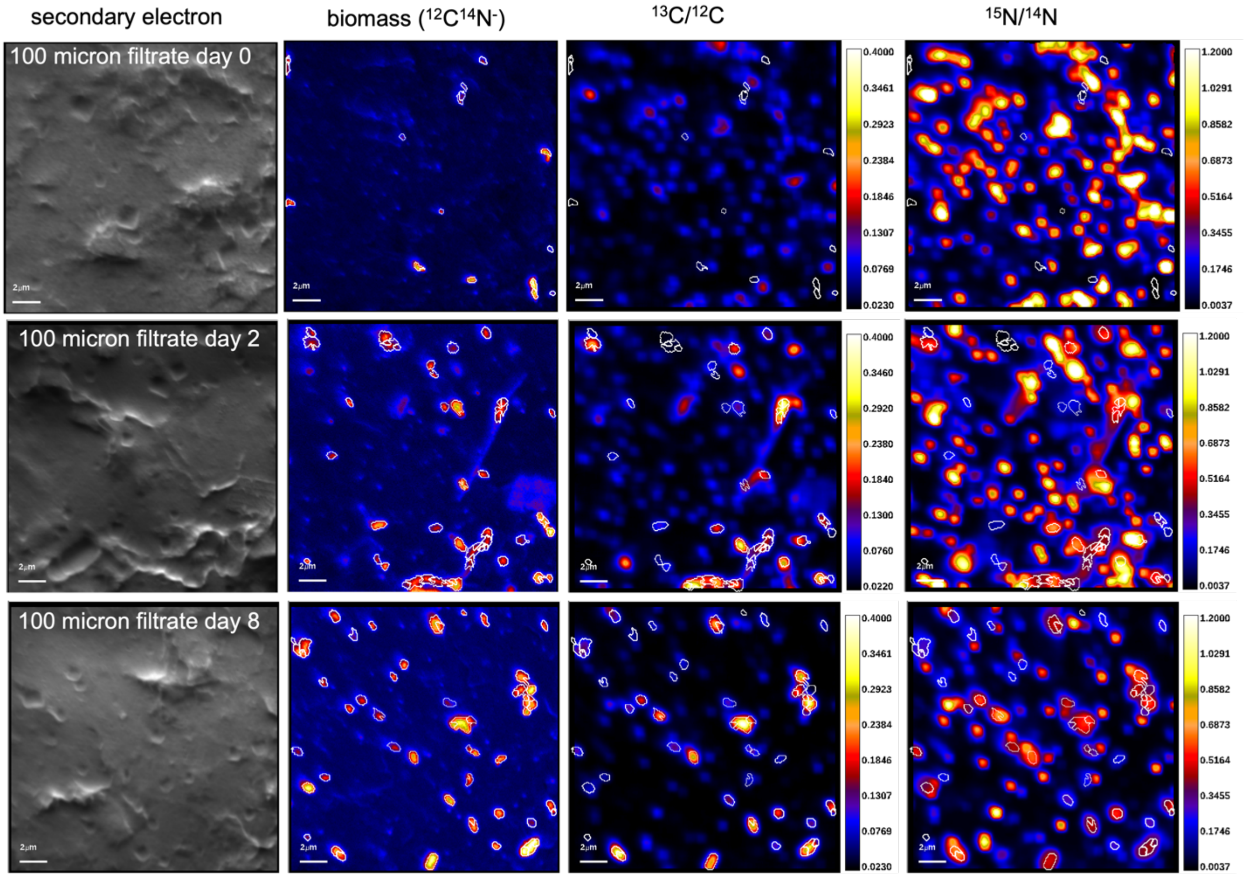

Figure S4: representative nanoSIMS images from the high purification experiment (#2). Regions of interests (ROIs, circled in white) for isotope quantification were chosen based on the $^{12}C^{14}N^-$ images that identify organic particles (the filter does not contain N). On day 0, the isotope labeled particles that represent the labeled EhVs generally do not correspond to those ROIs because the EhV particles have little $^{12}C^{14}N^-$ (they are mostly $^{12}C^{15}N^-$). After 2 and 8 days, some of the ROIs have become strongly isotope labeled, some are not, and there still remain many isotope labeled particles that do not correspond to ROIs (representing remaining EhVs).

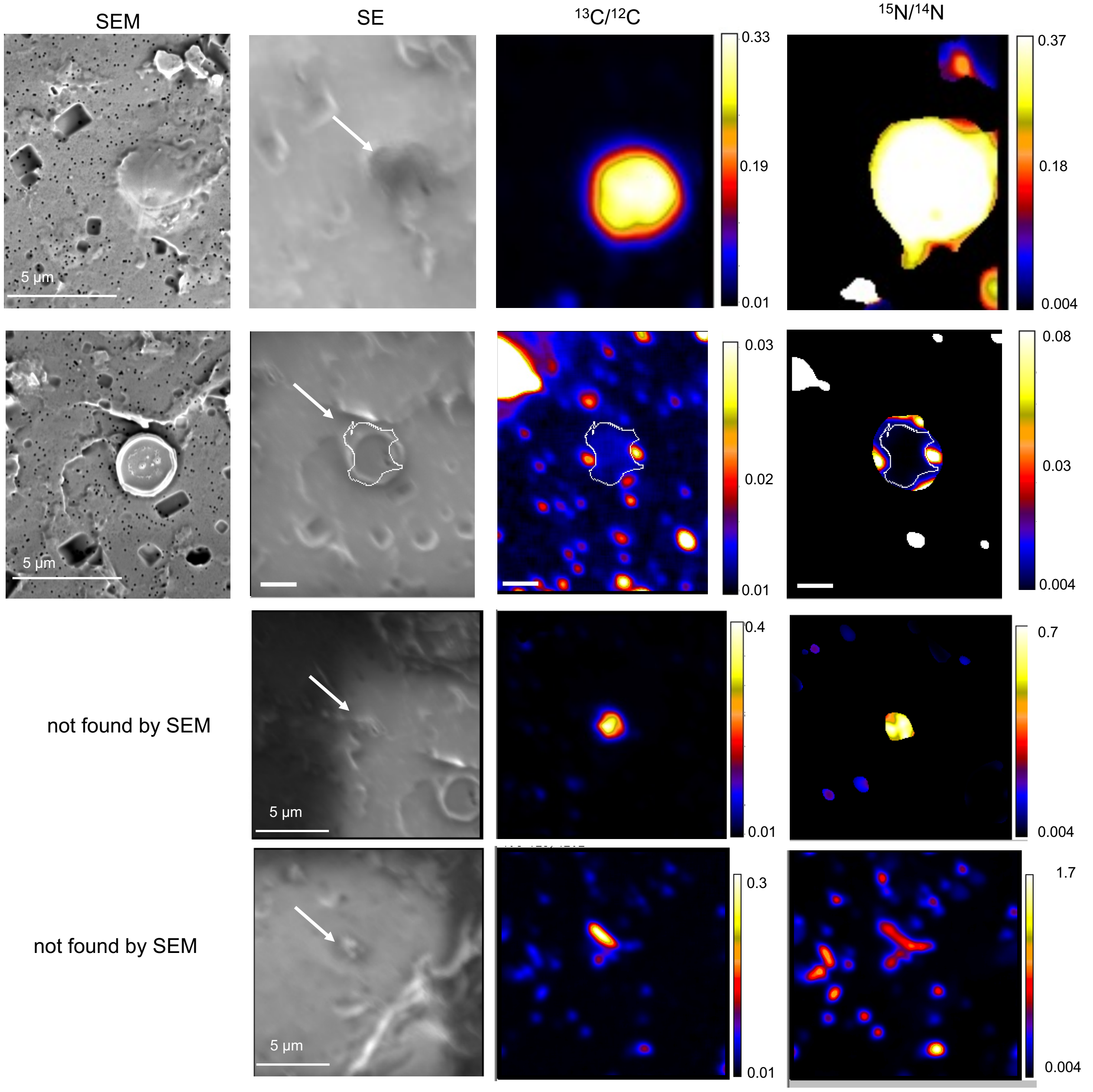

Figure S5: SEM and nanoSIMS images from protists identified in the high purification experiment sampled on day 2. The protist shown in the top panel was highly labeled in ^{13}C and ^{15}N. The protist shown in the second row was not isotopically enriched (the isotopically enriched hot spots next to the cell were likely viral particles or bacterial cells that became attached or were filtered underneath the protist cell; note the region of interest in white was drawn to avoid those hot spots). The bottom 2 rows (not found by SEM) show two smaller sized protists that were highly isotopically enriched.
